# Provision of acute care pathways for older major trauma patients in the UK

**DOI:** 10.1186/s12877-022-03615-1

**Published:** 2022-11-29

**Authors:** Heather Jarman, Robert Crouch, Mary Halter, George Peck, Elaine Cole

**Affiliations:** 1 Emergency Department Clinical Research Group, St George’s University Hospitals NHS Foundation Trust, Blackshaw Road, London, SW17 0QT UK; 2 Emergency Department, University Hospital Southampton NHS Foundation Trust, Tremona Road, Southampton, SO16 6YD UK; 3 Imperial College Healthcare NHS Trust, St Mary’s Hospital, Praed Street, London, W2 1NY UK; 4 Queen Mary University of London, 4 Newark Street, London, E1 2EA UK

**Keywords:** Multiple trauma, Pathway, Geriatrician

## Abstract

**Background:**

The introduction of specific pathways of care for older trauma patients has been shown to decrease hospital length of stay and the overall rate of complications. The extent and scope of pathways and services for older major trauma patients in the UK is not currently known.

**Objective:**

The primary objective of this study was to map the current care pathways and provision of services for older people following major trauma in the UK.

**Methods:**

A cross-sectional survey of UK hospitals delivering care to major trauma patients (major trauma centres and trauma units). Data were collected on respondent and site characteristics, and local definitions of older trauma patients. To explore pathways for older people with major trauma, four clinical case examples were devised and respondents asked to complete responses that best illustrated the admission pathway for each.

**Results:**

Responses from 56 hospitals were included in the analysis, including from 25 (84%) of all major trauma centres (MTCs) in the UK. The majority of respondents defined ‘old’ by chronological age, most commonly patients 65 years and over. The specialty team with overall responsibility for the patient in trauma units was most likely to be acute medicine or acute surgery. Patients in MTCs were not always admitted under the care of the major trauma service. Assessment by a geriatrician within 72 hours of admission varied in both major trauma centres and trauma units and was associated with increased age.

**Conclusions:**

This survey highlights variability in the admitting specialty team and subsequent management of older major trauma patients across hospitals in the UK. Variability appears to be related to patient condition as well as provision of local resources. Whilst lack of standardisation may be a result of local service configuration this has the potential to impact negatively on quality of care, multi-disciplinary working, and outcomes.

**Supplementary Information:**

The online version contains supplementary material available at 10.1186/s12877-022-03615-1.

## Background

Major trauma is a significant health burden for older patients. There is no universally accepted definition of major trauma, but the term is often used to denote serious injury that may result in permanent disability or death [1]. At patient level major trauma can describe injuries occurring in more than one body region, those with physiological compromise following injury or those that require admission to critical care [2, 3]. The UK national trauma registry (TARN) reported that between 2008 and 2017 there was a two-fold increase in major trauma in over 60s at over 8000 patients during the report period, accounting for more than half of severely injured patients in the UK [4]. Older people who sustain injury experience worse outcomes, higher mortality and poorer quality of life post-injury when compared to younger people, due to existing comorbidities, polypharmacy and frailty [5–7]. They are also more likely to have a longer hospital length of stay and have greater resources use on discharge [8, 9].

Previous studies have highlighted deficiencies in organisational and clinical aspects of major trauma care for older people leading to under-recognition of injury severity, delays in transfer for specialist care and delays to diagnostic imaging [10–12]. The need for focused and specialist care for older trauma patients has been suggested as a way to address these deficits with the development of a number of clinical standards, guidelines and recommendations [13–15]. In addition, the introduction of specific pathways of care for older trauma patients has been shown to decrease hospital length of stay and the overall rate of complications [16, 17].

Pathways are a means to organise care and defined generally as “a method for the patient-care management of a well-defined group of patients during a well-defined period of time” ([18], p562). They aim to standardise processes by giving the optimum sequence and timing of points or interventions in a patient’s care and can reduce variation and improve patient outcomes [19, 20]. In the US the introduction of standardised, evidence-based interdisciplinary pathway for trauma patients aged 65 and over (including screening, standardized order set and interdisciplinary input) significantly reduced 30-day readmission and rates of delirium [21]. Similar outcome improvements are reported in other studies where there is proactive involvement of a specialist geriatric care team as part of the care pathway [22, 23]. Components of these pathways vary but have a common approach focussing upon the early identification of injury in older major trauma patients and treatment of specific injuries, alongside aspects of comprehensive geriatric assessment such as management of delirium, cognitive impairment, nutrition, and medicines.

The UK National Institute for Health and Care Excellence (NICE) recommend that there are acute specialist services for older trauma patients but do not specify or provide guidance on how these are configured [1]. The extent and scope of pathways and services for older major trauma patients is not currently known. Therefore, the primary objective of this study was to map the current care pathways and provision of services for older people with major trauma in the UK.

## Methods

We conducted a UK-wide online cross-sectional survey to ascertain service provision and care pathways for older patients experiencing major trauma. The methods and results of this study are reported in line with the checklist for reporting results of internet E-Surveys (CHERRIES) [24]. All methods were performed in accordance with the relevant guidelines and regulations and with the Declaration of Helsinki. As this survey study contained no patient level data, and was distributed using professional collaborative networks, ethical approval from the Health Research Authority and Research Ethics Committee was not required (Supplementary material). Patients were not included in the survey; therefore formal consent to participate was not needed. By agreeing to participate in the survey informed consent by the responding health professional was presumed.

### Study sites

Study sites were any public UK hospitals delivering care to major trauma patients. In the UK, major trauma care is delivered in 27 regionalised networks responsible for patients within a geographical area [25]. Three tiers of hospital are designated based on resource availability: Major Trauma Centres (MTC), Trauma Units (TU) and local emergency hospitals (LEH) [26]. There are 25 MTCs in the UK similar to level 1 trauma centres elsewhere in the world, where specialist equipment and resources are available to provide care to the most severely injured patients 24 hours a day [27]. TUs (level 2–3 centres) have facilities to provide immediate resuscitation but do not have specialist services such as neurosurgery, and cannot provide definitive treatment for multiply injured patients [25, 28]. LEHs are part of trauma networks and do not routinely receive major trauma patients; these were included in our sample due to the blurred boundary for being defined as ‘major’ trauma in older people [27].

### Survey development

An internet-based questionnaire was developed by a group of UK major trauma clinicians and geriatricians using Microsoft Forms (Microsoft Office 365 E3, Redmond, Washington, US). Data were collected on respondent and site characteristics and local definitions of older trauma. To explore major trauma pathways for older people, four clinical case examples (vignettes) were devised (Table 1). Vignettes are hypothetical scenarios that partially represent real-life situations to stimulate a response and are widely used to evaluate and identify variations in practice across systems [29, 30]. The vignettes were developed by GP and peer reviewed by a group of UK-based geriatricians to reflect ‘typical’ clinical presentations of older major trauma cases. Data on the first admitting service in their hospital for each case vignette was collected with respondents indicating the likelihood of admission under a range of clinical specialties. These were defined as “always” (patient would always be admitted under that service), “sometimes” (patient would sometimes be admitted under that service) and “rarely” (patient not usually or rarely admitted under that service). Respondents were asked to complete responses that best illustrated the next step in the pathway for each clinical case example in their organisation, with open text comments for further explanation if required. The final part of the survey explored availability of guidelines and resources specific to the management of older trauma patients.

**Table 1:** Case vignettes

Four cases representing a variety of trauma presentations in older patients.	
Case 1	83-year-old female presenting after a fall. Full trauma CT identifies the only injury as a minor frontal cerebral contusion. No other injuries. Mildly confused but no focal neurological deficit. Patient requires 24 hrs neurological observations but no neurosurgical intervention.
Case 2	68-year-old male presenting after fall 3 m from a ladder. Full trauma CT identifies isolated chest trauma (left 8th and 9th rib fractures but no pneumo-haemothorax). On apixaban for atrial fibrillation. No other injuries. He is in moderate pain after 5 mg morphine.
Case 3	91-year-old female mechanical fall getting out of the shower. Has dementia. Has home care four times a day. CT head and neck reveals a 3rd cervical vertebral fracture. Neurosurgical team advise conservative management with Miami J collar
Case 4	72-year-old male fall 14 steps. Initially had acute right sided subdural haemorrhage requiring craniotomy and evacuation of haematoma. Slow improvement on ICU and remains with a tracheostomy and nasogastric tube. Awaiting a percutaneous endoscopic gastrostomy and having tracheostomy weaning. He is ready for stepdown from intensive care unit / ready for repatriation to his local trauma unit.

### Recruitment process and survey administration

The survey was open from October to December 2020. An invitation to participate was circulated to clinical leads in MTCs, TUs or LEHs through trauma network managers and trauma clinical service leads with one follow-up reminder sent. A survey link was posted on social media. A short invitation to participate was included at the start of the survey which was six screens (pages) in length consisting of 20 mandatory questions. Open comment sections were not mandated for completion. Respondents were able to review their responses prior to submission. Completion was voluntary with no incentives offered.

Data were exported for analysis to Microsoft Excel (Microsoft Office 365 E3, Redmond, Washington, US). Where duplicate responses were received from the same organisation the response included in the analysis was selected from the role likely to carry the with greatest knowledge of the trauma pathways in their hospital. Responses were prioritised in the following order: 1. geriatrician / orthogeriatrician; 2. major trauma clinical leads (leading the major trauma service in the hospital); 3. trauma coordinator (overseeing the major trauma patients in the hospital); 4. other. Duplicate responses from organisations were removed prior to analysis. Responses were anonymised with an identification number.

### Data analysis

Descriptive statistics were used to provide frequency (counts, percentages and cross-tabulation) of fixed choice responses. Open responses were analysed from all returned surveys. Free-text responses that formed part of an ‘other’ option in the survey, for instance specialty responsible for admitting a patient, were included in the descriptive statistics. Where respondents provided additional free-text information relating to the pathway of care for patients detailed in the case vignettes, these were analysed using a thematic content approach [31, 32]. Free-text responses were pulled from the data, listed and initially coded into themes by one researcher (HJ). A second researcher (EC) independently verified the coding. Both researchers then discussed and reviewed the emergent themes. Illustrative verbatim quotes were extracted.

## Results

### Respondents

**Fig. 1:**
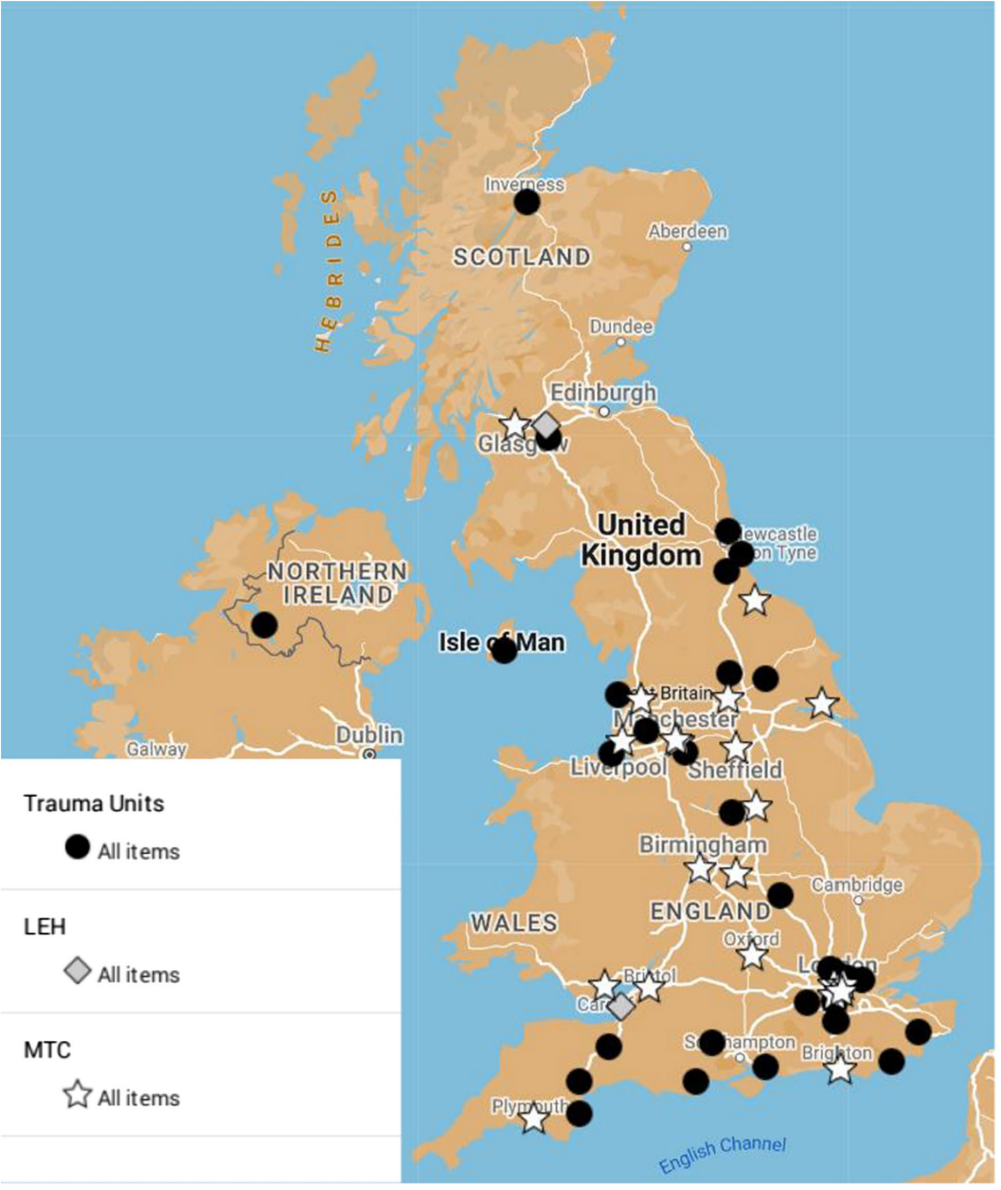
Map data (Copyright) GeoBasis-DE/BKG (copyright 2009), Google UK map showing respondents by location and hospital type

Eighty-two responses were received. Four responses received from outside of the UK and one with no hospital name provided were excluded. Following removal of duplicates, responses from 56 different hospitals were included in the analysis. Of 25 MTCs receiving adult patients in the UK (21, 84%) were represented plus 33 TUs and two LEHs (Fig. 1). Responses were received from at least one hospital in all the trauma networks in England and Wales, two of the four trauma networks in Scotland and the trauma network in Northern Ireland.

### Age definition for ‘geriatric’ or ‘older’ within the hospital

The majority of responses (40, 73%) defined ‘old’ by chronological age, less than 10% did not have a definition and the remainder using frailty-based definitions or a combination of age with a qualifying criterion (frailty or care home residency). The most frequently reported definition was 65 years and over (26, 46%) and this did not differ greatly between MTCs, TUs and LEHs.

### Process characteristics

#### Hospital guidelines for older major trauma patients

The availability of specific guidelines for older major trauma varied across hospitals, with 283 available guidelines reported in total. All but one centre had at least one guideline in place. Specific guidelines for falls referral comprised almost half (27, 48%), followed by rib fracture management (24, 43%). Combined guidelines, where older people were included in protocols for all adult trauma patients, predominated (Table 2).

**Table 2:** Availability of older major trauma guidelines

		Older patient specific trauma guidance				Combined older and adult trauma guidance			
		Total *n* = 56	Major Trauma Centre *n* = 21	Trauma Unit *n* = 33	Local Emergency Hospital *n* = 2	Total *n* = 56	Major Trauma Centre *n* = 21	Trauma Unit *n* = 33	Local Emergency Hospital *n* = 2
Trauma triage guideline	n	17	8	9		25	8	17	
	%	30%	38%	27%		45%	38%	52%	
Trauma call activation criteria	n	14	8	6		28	9	19	
	%	25%	38%	18%		50%	46%	58%	
Trauma imaging guideline	n	13	5	8		27	15	11	1
	%	23%	24%	24%		48%	71%	33%	50%
Trauma admission clerking proforma	n	9	4	5		30	9	21	
	%	16%	19%	15%		54%	46%	64%	
Falls referral guideline	n	27	10	16	1	11	3	8	
	%	48%	48%	48%	50%	20%	14%	24%	
Rib fracture management guideline	n	24	9	15		29	12	16	1
	%	43%	46%	45%		52%	57%	48%	50%
Rehabilitation guideline	n	5	2	3		24	8	16	
	%	9%	10%	3%		43%	38%	48%	
**Total guidelines**	**n**	**109**	**46**	**62**	**1**	**174**	**64**	**98**	**1**

#### First admitting specialty

**Fig. 2:**
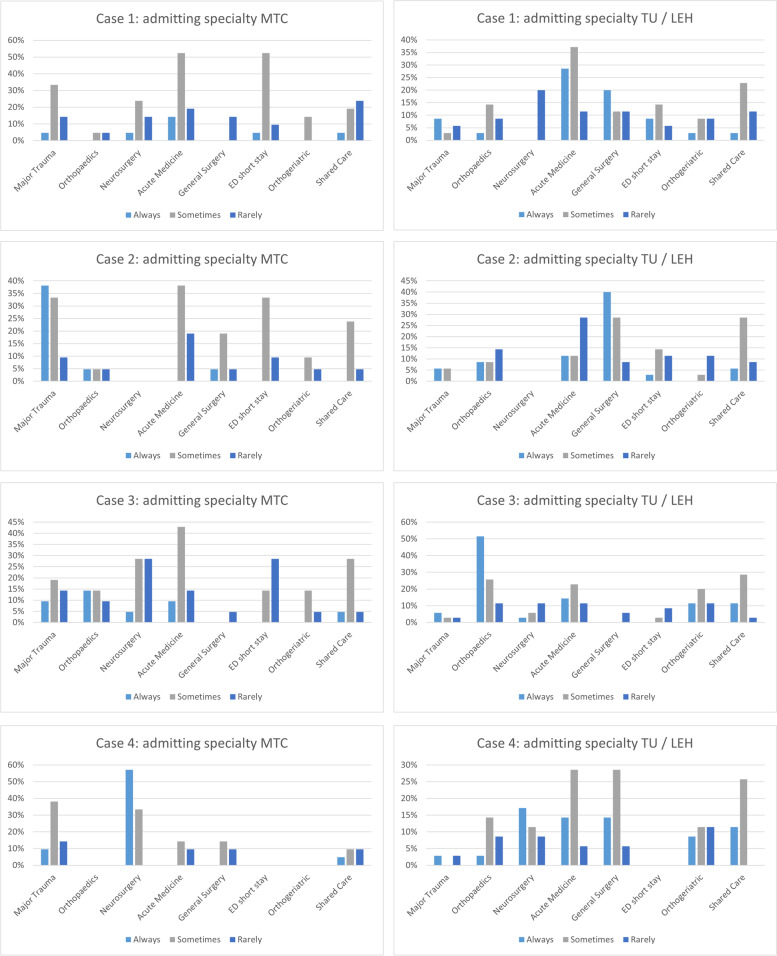
Summary of admitting service in MTC (*n* = 21) and TU/LEH (*n* = 35) by case for each of the case vignettes. Categories of always, sometimes or rarely. Legend: Case 1 (patient with cerebral contusions); Case 2 (patient with rib fractures); Case 3 (patient with cervical spine injuries); Case 4 (step-down / repatriation patient with subdural haematoma)

Older major trauma management involves a wide range of specialties and professions. The primary admitting specialty varied across cases and locations of care (Figs. 2). Acute medical and surgical specialities predominated in admitting TU patients following traumatic brain injury (Fig. 2, case 1 and 4) whereas ED short stay areas and neurosurgery were more likely to admit these patients in MTCs (Fig. 2). Orthogeriatricians always admitted the majority of rib injured patients (case 2) in both levels of care.

#### Frailty and geriatrician assessment

**Fig. 3:**
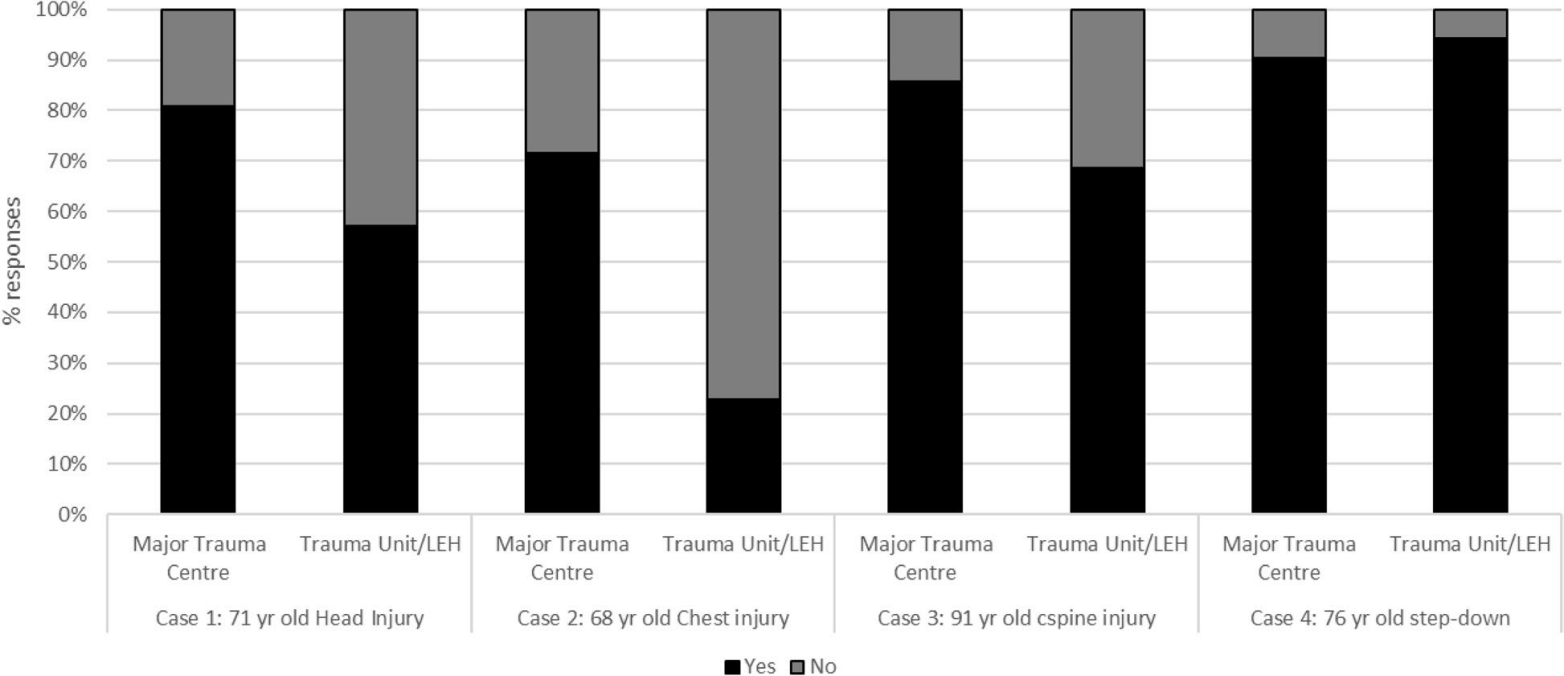
Cases seen by geriatrician. Legend: Yes = seen by a geriatrician, No = not seen by a geriatrician

Over two thirds of respondents (39, 70%) indicated that patients were routinely assessed for frailty in the emergency department (ED), and this was similar for MTCs and TUs. The most common tool was the Clinical Frailty Scale (36 of 39 respondents, 92%). Assessment by a geriatrician varied and was associated with increased age or length of time in hospital. For example, 75% (42) of respondents suggested a 72-hour geriatric review would be achieved in vignette 3 (91-year-old with dementia), whereas only 41% (23) for the 68-year-old with isolated chest injuries (vignette 2). The patient requiring step-down following intensive care was mostly likely to receive a geriatrician review in both TU and MTC (52, 93%). Levels of geriatrician review were higher in MTCs than in TUs, except for the patient requiring step-down from ICU where they were more likely to be reviewed in the TU than the MTC (Fig. 3).

### Open responses

Open responses revealed complexities in defining pathways for older major trauma patients with two themes predominating: a) organisational factors influencing decisions; and b) the role of the geriatrician.

Under the theme of ‘organisational factors influencing decisions’ respondents indicated that patients would often experience variation in their care that was dependent on organisational factors such as availability of beds or day of the week rather than due to the patient’s clinical needs, for example:

“Often these patients will have a prolonged stay on our Acute Medical Unit (AMU) due to difficulties agreeing under which specialty the patient will be admitted.” (Vignette 1 traumatic brain injury; Major Trauma Centre respondent).

“We are now being pressured to take these to general frailty wards where nurses have had training.” (Vignette 2 chest injury; Trauma Unit respondent).

“We have a traumatic brain injury (for both injury and medical pathology) rehabilitation ward on site, but capacity is limited, so if patient not able to transferred directly would come in under care of medical team whilst awaiting a bed on the TBI unit” (Vignette 4 ICU step down; Trauma Unit respondent).

The case example of a patient with a mild traumatic brain injury elicited the greatest number of responses regarding pathway variation, with patients being admitted variously under general surgery, medicine, emergency short-stay areas and stroke teams.

“Usually admitted to neurosurgery. However depends who is on-call!” (Vignette 1 traumatic brain injury; Major Trauma Centre respondent).

“If she was deemed to be frail, a discussion would be had by ED with the Frailty team and if they agreed she might be admitted under them rather than general surgery. We are keen to develop a pathway where this lady would be admitted to our major trauma ward under the neurosurgeons with access to a trauma geriatrician but are some way off managing that (due no current funding for a trauma geriatrician (due soon...) and challenges around appropriate beds.” (Vignette 1 traumatic brain injury; Major Trauma Centre respondent).

“Isolated head injuries should be admitted under medicine. They may go on to a geriatric ward, acute medical ward or the stroke ward dependent on bed availability. (Vignette 1 traumatic brain injury; Trauma Unit respondent).

Under the second theme, titled ‘the role of the geriatrician’, different models of geriatrician input were evident across both MTCs and TUs, with staffing levels impacting on some teams’ ability to review patients:

“Due to reduced staffing as a result of retirement and staff moving to another trust, the orthogeriatrics team had to reduce their service. Last year, the patient would be admitted under joint orthogeriatric/orthopaedic care and had a geriatrician review within 72 hours, but not now.” (Trauma Unit respondent).

“Currently no routine ability to be seen by geriatrician.” (Major Trauma Centre respondent).

“Due to staffing constraints within orthogeriatrics, we are unable to now routinely see these patients.” (Trauma Unit respondent).

“Frailty Trauma ward round is Mon/Wed/Fri so would not be seen by geriatrician if admitted Monday evening and discharged before Wednesday ward round” (Major Trauma Centre respondent).

In contrast there was evidence of clear geriatrician involvement in the care of older major trauma patients by some respondents.

“Would be automatically reviewed by frailty team if admitted to acute medicine and ortho-geriatrician if admitted under orthopaedics.” (Trauma Unit respondent).

“The ED team would refer to the MTC neurosurgeons for advice and then the patient would be admitted under the acute medical take team They would subsequently be handed over to the care of the elderly team with ongoing advice from the neurosurgeons at the MTC or input from general surgery as needed.” (Trauma Unit respondent).

“New pathway live as of last month for shared care for spinal surgery review in ED, prescription of collar and spinal precautions and then admission under Geri [atrician] for shared care.” (Major Trauma Centre respondent)

## Discussion

The results of this survey illustrate the significant variation in the pathways of care and management of older major trauma patients in the UK which appears to be dependent on patient condition and local service provision. Whilst older trauma patients are a heterogeneous group, they typically have decreased physiological reserve and conditions that pose challenges for management and clinical decision making. The heterogeneity of this group was evident in how the various organisations defined ‘older’ in relation to their services. There was lack of consensus as to which patients would be classified as old, with the chronological age ranging from 60 to 82 years. This inability to provide a strict definition is reflected in both policy and research with ‘old’ defined variously as starting at 55 years [33], 60 years [34] or 65 years [35]. A systematic review and meta-analysis of geriatric consults similarly found variation in the age cut-offs to define this patient group [36]. The aging process is not uniform and there is increasing evidence that it is frailty, rather than age, that impacts on outcomes in trauma patients [6, 37, 38]. This has led to increasing consideration of identifying frailty early in the patient admission with recommendations in guidance for emergency care and trauma settings. In UK, there is guidance that clinical frailty assessment should be completed within 30 minutes of arrival into the Emergency Department [39]. Whilst this indicator does not apply to TUs, a high proportion of this level of hospital (69%) routinely assess for frailty in older trauma patients within the ED, with some using frailty to differentiate need for geriatrician assessment. The majority used the Clinical Frailty Scale [40] which is shown to be feasible and accurate to undertake in older major trauma patients in the ED [41].

Clinical practice guidelines aim to support clinicians to make informed decisions about appropriate care, and there is evidence to suggest the standardisation of care of older major trauma patients improves outcomes [42, 43]. The physiological, psychological and social changes associated with ageing require specialised integrated care for older people and in the UK this has been repeatedly highlighted in professional and clinical guidelines [1, 13, 44]. Whilst this survey provides evidence of pathways and protocols for older people’s trauma care being in place, the challenges moving forward are to identify which of the available guidance impacts positively on experience and outcome, and to validate patient centred outcomes for this population of patients.

The responses to the case vignettes indicate there is some attempt to organise the care for older major trauma patients, but this varied widely across organisations. The case vignettes used in this survey were designed to represent elements of the pathway for patients who are typically managed within the trauma system. Although there is crossover in the nomenclature, in contrast to guidelines which usually focus on a single condition or aspect of care, clinical pathways are defined as “a complex intervention for the mutual decision making and organization of care for a well-defined group of patients during a well-defined period” [45]. They aim to translate clinical evidence into local practice and may be associated with reduced in-hospital complications, decreased length of stay and hospital costs [46, 47]. Our results show patients with the same injury pattern and needs being managed differently between and within MTCs and TUs. The Trauma Units appeared to report more instances where decisions relating to admission and treatment were influenced by clinical service organisation rather than patient need. The differences in the configuration of services and availability of resources between MTCs and TUs may go some way to explain this variation and, considering the heterogeneity in older major trauma, legitimate deviation from pathways may be necessary when deemed clinically appropriate for individual patients. However, respondents indicated that in some areas the management of patients varied according to staffing levels, day of the week and individual clinician preference. Although best practice models of trauma care for older people have been described, gaps in service provision that lead to divergence from these models have also been identified [48, 49] and the impact of this requires further investigation.

The most marked variability in our survey was in the admitting specialty. There is requirement for UK MTCs to admit major trauma patients under a defined team with a designated consultant, usually called the major trauma service [1, 27]. The patients in this survey all represent those with ‘major trauma’ but respondents indicated they would not always be admitted under the major trauma service. Guidance for TUs is less stringent, recommendations exist for management of older major trauma patients in a designated pathway which includes a coordinated multidisciplinary approach [15].

Within all vignettes a proportion of respondents reported a model of ‘shared care’ or ‘joint care’. Although these arrangements were not fully described, shared care most often refers to patient admission under a primary clinical specialty supported by geriatric medicine. These models are widely used in hip fracture patients as ‘orthogeriatric care’ and are associated with lower mortality compared to standard care models [50, 51]. Attention has focused on the need to provide geriatrician input into the care of older major trauma patients [13, 44], although the optimum model is not known. Several examples of major trauma geriatrician models have been proposed with positive effects on length of stay, in-hospital complications and discharge destination [17, 22, 52]. Recently a payment subsidy has been introduced within the UK, requiring patients aged 65 and over to have a frailty assessment completed within 72 hours of admission to an MTC by a geriatrician [35]. In our survey, not all of the trauma patients in the case vignettes would be routinely reviewed by a geriatrician in a MTC despite presenting under a ‘major trauma’ pathway. There was evidence that geriatrician review would be carried out in some TUs, most commonly after 72 hours. Given the limited resource available, further work is required to determine which older trauma patients benefit most from early geriatrician involvement.

### Limitations

There are several limitations to this survey. First, the survey was sent to existing networks and supplemented by dissemination through social media channels which produced duplicate responses from organisations and non-response from others. Despite this, respondents provided data that reflected both positive and negative aspects of care within their hospitals that does provide some balance to this argument. The 84% response rate suggests the data from the MTCs is representative. Despite Trauma Units receiving most trauma patients over 75 years of age in the UK [TARN], we acknowledge that the participation rates from Trauma Units and Local Emergency Hospitals was low compared to MTCs and therefore this group is under-represented. It is possible that those hospitals who did not respond were less likely to be following national guidance and did not wish to provide information, suggesting that non-response bias could lead to an under-estimation of the problem in UK hospitals.

The use of case vignettes, whilst stimulating responses for ‘typical’ trauma pathways are not able to fully capture the nuances of individual pathways or patients and therefore did not elicit responses regarding access to all services in the major trauma pathway such as physiotherapy or psychological support. We did not analyse guidelines themselves so did not assess for quality or relevance. Finally, as this survey measures current UK practice it may not be applicable to all major trauma settings, although may be of interest to those with similar trauma systems.

## Conclusion

 This survey highlights wide variability in the admission specialty and subsequent management of older major trauma patients across hospitals in the UK. Pathways and protocols exist for older people’s trauma care, but they appear to be created for individual centres, open to interpretation and their delivery impacted by wider contextual and resource factors. Whilst lack of standardisation may be a result of local service configuration this has the potential to impact negatively on quality of care, multi-disciplinary working, and outcomes. Further understanding of the factors that influence the variation in practice is required and we would recommend a standardized approach to the development and implementation of care pathways for older trauma patients.

## Supplementary Information

**Additional file 1.** 

## Data Availability

The datasets generated and/or analysed during the current study are not publicly available as consent was not received by participants to use the data in the way but are available from the corresponding author on reasonable request.
