# Supplementary material for: Provision of acute care pathways for older major trauma patients in the UK
Source: BMC Geriatr. 2022 Nov 29;22:915. doi: 10.1186/s12877-022-03615-1 (PMC9706856; doi:10.1186/s12877-022-03615-1)
Supplement: Supplementary file 1 — Additional file 1. [file 12877_2022_3615_MOESM1_ESM.docx]

# **SUPPLEMENTARY MATERIAL**

**Pathways for Older Major Trauma patients: a survey of UK practice**

| What is the name of your hospital? | Short answer |
| --- | --- |
| Is your hospital a:   - Major Trauma Centre - Trauma Unit - Local emergency hospital | Fixed choice |
| . Are you a?   - Doctor - Nurse - AHP - MTC or Network manager - Other (please specify) | Fixed choice |
| What is your job title? | Short answer |
| What is the age definition for ‘geriatric’ within your hospital/Trust? If there is more than one please do expand. | Short answer |
| SECTION BREAK | |
| The following case examples represent a variety of trauma presentations in older patients. Please read them carefully and indicate in the questions following the *typical* pathway they would take in your hospital. We appreciate that there may not be an exact fit for some of the case examples so there is a ‘freetext’ space with each scenario if you wish to elaborate. | |
| Case 1: 83 yr female presenting after a fall. Has full trauma CT. Only injury identified is a minor frontal cerebral contusion. No other injuries. Mildly confused but no focal neurological deficit. Requires 24hrs neuro observations from a neurosurgical perspective. | |
| In your hospital, under which team would this patient most likely be admitted:  Major trauma specific specialty team  Orthopaedics  Neurosurgery or if a TU/district general hospital transfer to the local neurosurgical unit  Acute medicine  Acute surgery  Emergency Department (i.e short stay or observation unit)  Orthogeriatrics  Shared care (please expand) (freetext) | Fixed choice with free text option |
| Would they routinely be seen by a geriatrician if admitted for:  Less than 72 hours  More than 72 hours | Fixed choice |
| Would they have access to a specialist neuro-therapy services?  Yes  No  Not available in my hospital | Fixed choice |
| Please add any other comments about the pathway of care this patient might take in your hospital | Freetext |
| Case 2: 68-year-old male presenting after fall 3m from a ladder. Full trauma CT identifies isolated chest trauma (left 8^th^ and 9th rib fractures but no pneumo-haemothorax). On apixaban for atrial fibrillation. No other injuries. He is in moderate pain after 5mg morphine. | |
| In your hospital, under which team would this patient most likely be admitted:  Major trauma specific specialty team  Orthopaedics  Neurosurgery or if a TU/district general hospital transfer to the local neurosurgical unit  Acute medicine  Acute surgery  Emergency Department (i.e short stay or observation unit)  Orthogeriatrics  Shared care (please expand) (freetext) | Fixed choice with free text option |
| Would they routinely be seen by a geriatrician if admitted for:  Less than 72 hours  More than 72 hours | Fixed choice |
| Would they have access to an acute pain service review?  Yes  No  Not available in my hospital | Fixed choice |
| Does your trust have guidelines for rib fracture management specific for older people?  Yes, specifically for older people  Yes, but not specifically for older people  No | Fixed choice |
| Please add any other comments about the pathway of care this patient might take in your hospital | Freetext |
| Case 3: 91-year-old female mechanical fall getting out of the shower. Has dementia. Has home care four times a day. CT head and neck reveals a 3rd cervical vertebral fracture. Neurosurgical team advise conservative management with Miami J collar | |
| In your hospital, under which team would this patient most likely be admitted:  Major trauma specific specialty team  Orthopaedics  Neurosurgery or if a TU/district general hospital transfer to the local neurosurgical unit  Acute medicine  Acute surgery  Emergency Department (i.e short stay or observation unit)  Orthogeriatrics  Shared care (please expand) (freetext) | Fixed choice with free text option |
| Would they routinely be seen by a geriatrician if admitted for:  Less than 72 hours  More than 72 hours | Fixed choice |
| Would they have access to an acute pain service review?  Yes  No  Not available in my hospital | Fixed choice |
| Please add any other comments about the pathway of care this patient might take in your hospital | Freetext |
| Case 4: 72-year-old male fall 14 steps. Initially had acute right sided subdural haemorrhage requiring craniotomy and evacuation of haematoma. Slow improvement on ICU and remains with a tracheostomy and nasogastric tube. Awaiting a percutaneous endoscopic gastrostomy and having tracheostomy weaning. He is ready for stepdown from intensive care unit / ready for repatriation to his local trauma unit. | |
| When stepped down from ICU/repatriated to your hospital, under which team would this patient most likely be admitted under:  ICU/HDU (asthe only place tracheostomies are managed in our hospital)  Major trauma specific specialty team  Orthopaedics  Neurosurgery or if a TU/district general hospital transfer to the local neurosurgical unit  Acute medicine  Acute surgery  Emergency Department (i.e short stay or observation unit)  Orthogeriatrics  Shared care (please expand) (freetext) | Fixed choice with free text option |
| Would they have access to a specialist neuro-therapy services?  Yes  No  Not available in my hospital | Fixed choice |
| Would they routinely be seen by a geriatrician ? | Fixed choice |
| Please add any other comments about the pathway of care this patient might take in your hospital | Freetext |
| SECTION BREAK | |
| What resources are available in your hospital for older trauma patients: | Choice:  Yes, specific to older people  Yes, included in combined guideline or protocol  Not available  Don’t know |
| Trauma triage guidance |  |
| Trauma call activation criteria |  |
| Trauma imaging guideline |  |
| Trauma admission clerking / screening guideline |  |
| Falls referral guideline |  |
| Treatment escalation or end of life care guideline |  |
| Rehabilitation guideline |  |
| Do you routinely assess frailty in the ED for older major trauma patients? | Fixed choice |
| Which tool do you use for assessment of frailty in the ED?  Clinical Frailty Scale (CFS)  PRISMA-7  FRAIL scale  Trauma specific frailty index (TSFI)  Other (freetext) |  |
| END | |
